# Temperature Shift Experiments Suggest That Metabolic Impairment and Enhanced Rates of Photorespiration Decrease Organic Acid Levels in Soybean Leaflets Exposed to Supra-Optimal Growth Temperatures

**DOI:** 10.3390/metabo5030443

**Published:** 2015-08-05

**Authors:** Richard C. Sicher

**Affiliations:** Crop Systems and Global Change Laboratory, United States Department of Agriculture-Agricultural Research Service, Room 332, Bldg. 001, BARC-west 10300 Baltimore Avenue, Beltsville, MD 20705, USA; E-Mail: richard.sicher@ars.usda.gov; Tel.: +1-301-504-6632; Fax: +1-301-504-5823

**Keywords:** primary metabolism, TCA cycle, photosynthetic flux, CO_2_ enrichment, abiotic stress, leaf development

## Abstract

Elevated growth temperatures are known to affect foliar organic acid concentrations in various plant species. In the current study, citrate, malate, malonate, fumarate and succinate decreased 40 to 80% in soybean leaflets when plants were grown continuously in controlled environment chambers at 36/28 compared to 28/20 °C. Temperature effects on the above mentioned organic acids were partially reversed three days after plants were transferred among optimal and supra-optimal growth temperatures. In addition, CO_2_ enrichment increased foliar malate, malonate and fumarate concentrations in the supra-optimal temperature treatment, thereby mitigating effects of high temperature on respiratory metabolism. Glycerate, which functions in the photorespiratory pathway, decreased in response to CO_2_ enrichment at both growth temperatures. The above findings suggested that diminished levels of organic acids in soybean leaflets upon exposure to high growth temperatures were attributable to metabolic impairment and to changes of photorespiratory flux. Leaf development rates differed among temperature and CO_2_ treatments, which affected foliar organic acid levels. Additionally, we report that large decreases of foliar organic acids in response to elevated growth temperatures were observed in legume species.

## 1. Introduction

Various adaptive mechanisms make it possible for terrestrial plants to acquire thermotolerance and to survive high and acutely high growth temperatures [1,2]. Managing heat stress, especially under field conditions, involves complex processes that stabilize membranes and proteins in order to preserve cellular function. First, heat stress induces reactive oxygen species (ROS), which oxidize cellular constituents and damage membrane integrity [3]. Consequently, the control of ROS is an important strategy in attaining thermotolerance. Second, specific proteins, known as heat shock proteins (HSPs), are synthesized in response to elevated growth temperatures. The role of HSPs is to protect functional proteins by preventing or delaying their denaturation or degradation [4,5]. Third, many plant metabolites alleviate environmental stress by functioning as osmolytes or compatible solutes and by assisting in the removal of ROS [6,7,8]. Elevated growth temperatures also affect rates of important metabolic processes, including photosynthetic CO_2_ assimilation, dark respiration and photorespiration [9]. Consequently, acclimation to enhanced growth temperatures involves wholesale changes of plant metabolism.

Soybean is a robust crop plant that is moderately tolerant of elevated growth temperatures, particularly during vegetative growth [10]. In a previous study [11], it was shown that 28 of 43 total leaf metabolites were altered in soybean leaflets when the growth temperature was increased from 28/20 to 36/28 °C. Net rates of CO_2_ assimilation were about 20% greater in the higher compared to the lower growth temperature treatment. Leaf starch decreased [12] and there was a shift in the accumulation of nonstructural carbohydrates from free sugars to polyols [13]. Raffinose and γ-aminobutyric acid (GABA) also increased but the majority of organic and amino acids present in leaflets decreased in response to elevated growth temperatures. Several organic acids associated with the tricarboxylic acid (TCA) cycle, most notably citrate, decreased by 50 to 90% in response to enhanced growth temperatures. The impact of elevated growth temperatures on organic acid levels was mitigated by growing soybean plants at twice ambient CO_2_ partial pressures [11,14]. At this time, it is not clear why TCA cycle intermediates decrease in response to elevated growth temperatures or why CO_2_ enrichment affected this process. It is also not clear how a temperature dependent down regulation of the TCA cycle affected other aspects of plant metabolism.

The objective of the current study was to determine whether temperature effects on TCA cycle intermediates and related organic acids in soybean leaflets were reversed by reciprocal transfers among optimal and elevated growth temperature treatments. We assumed that adjustments of leaflet organic acid concentrations that occurred over a period of minutes to hours were likely attributable to altered rates of net photosynthesis (P_N_) or photorespiration (P_R_). Alternatively, changes of organic acid levels that occurred several days after a growth temperature shift were more likely due to temperature dependent metabolic impairment.

## 2. Materials and Methods

### 2.1. Plant Materials

Experimental approaches and the equipment used in this study were as described previously [11], although important modifications are noted below. Measurements were normally performed on third trifoliolate leaflets of soybean seedlings (*Glycine max* L. [Merr.] cv. Kent) grown from single seeds in controlled environment chambers (model M-18, Environmental Growth Chambers Corp., Chagrin Falls, OH, USA). Additional comparative measurements were performed using common bean (*Phaseolus vulgaris* L. cv. Burpee’s Tenderpod), maize (*Zea mays* L. cv. Silver Choice) and pepper (Capsicum annum L. cv. Bugang) in the ambient CO_2_ treatment. Seeds were sown in 3 L plastic pots filled with vermiculite and air temperatures were either 28/20 or 36/28 °C on a light/dark basis. The photoperiod employed a 14 h/10 h light/dark cycle and the irradiance was 900 ± 35 μmol m^−2^s^−1^ (photosynthetic photon flux density). Chamber air CO_2_ partial pressures were controlled at 39 ± 2 and 70 ± 2 Pa as described previously [15]. Experiments with ambient and elevated CO_2_ were alternated between plantings using two of the above mentioned growth chambers. Temperature treatments also were cycled among chambers on different plantings. Pots were flushed daily with a complete mineral nutrient solution containing 14.5 mM total N. Relative humidity was between 60 and 80% during the light period and was usually 5 to 10% higher in the high compared to the low temperature treatment.

Experiments were normally initiated 21 or 22 d after sowing by reciprocally transferring five to six plants between chambers having differing growth temperature treatments. However, experiments with pepper were initiated 44 or 45 d after sowing due to slow growth at the supra-optimal temperature. Soybean harvests were initiated about 24 h after the third trifoliolate leaf attained full expansion in the 36/28 and 28/20 °C temperature treatments, respectively. The date that experiments were initiated was labeled day 0 and harvests were continued daily for 3 d. Four temperature treatments were employed. These were the 36/28 °C, 28/20 °C, and the two treatments obtained by transferring plants among temperature treatments. For soybean, three or four leaf discs (2.5 cm^2^ each) were harvested from third trifoliolate leaflets between 4 and 6 h after the start of the light period in all four temperature treatments. This was to minimize the impact of diurnal effects on leaflet metabolite levels. Leaf discs also were collected from pepper, maize and *Phaseolus* using the most recently, fully expanded leaf. Sampled tissue was immediately placed into 3 to 4 ml of liquid N_2_, transferred to labeled envelopes and lyophilized. Freeze dried samples were then stored for less than a month in a laboratory freezer at −20 °C until extracted.

### 2.2. Quantification of Leaf Components

Organic acids were extracted and analyzed as described previously [11,15,16]. Lyophilized leaf tissue (~30 mg DW) was pulverized to a fine powder and extracted after the methods of Roessner *et al.* [17]. Following derivatization with MSTFA (*N*-Methyl-*N*-(trimethylsilyl) trifluoracetamide), twelve specific organic acids were separated and identified using gas chromatography coupled to mass spectrometry. Quantitation was based on four point curves prepared with known chemical standards and peaks were accurately identified by mass spectra.

### 2.3. Statistical Comparisons

Experiments in this study were performed twice and, when necessary, a third experiment was performed to correct observed inconsistencies among specific metabolites in the first two experiments. Means and standard errors were determined for four samples from each experiment and average values were obtained by combining results of both experiments. Significant differences were determined using a two-way Analysis of Variance procedure (StatView 5.0, Mountain View, CA). Organic acid values were independent variables and harvest dates, chamber air temperatures and CO_2_ treatments were dependent variables (Supplementary Table S1).

## 3. Results

### 3.1. Growth Temperature Effects on Foliar Organic Acids in Ambient CO_2_ Treatment

**Figure 1:**
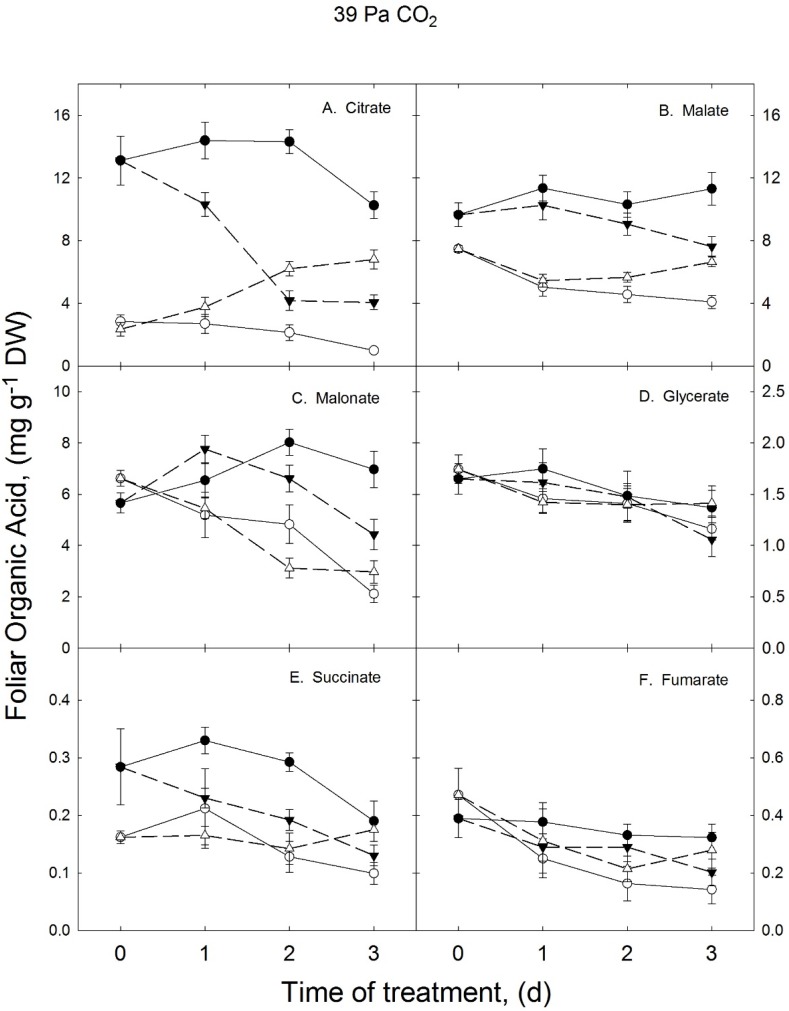
Effects of reciprocal changes of growth temperature on concentrations of organic acids in soybean leaflets grown with ambient CO_2_ partial pressures. Plants were grown from seeds using continuous growth temperatures of 28/20 °C (●—●) or 36/28 °C (○—○). The first 21 or 22 DAS selected plants were transferred from the higher to lower (∆-----∆) or from the lower to higher (▼-----▼) growth treatment.

Figure 1 shows effects of both continuous and reciprocal changes of nearly optimal (28/20 °C) and enhanced growth temperatures (36/28 °C) on six organic acids, including four TCA cycle intermediates, from soybean leaflets using plants grown with ambient CO_2_ (39 Pa) Citrate, malate and malonate were the most abundant organic acids in soybean leaf tissue (Figure 1A–C, respectively). Foliar citrate concentrations were 83% lower in the 36/28 *versus* the 28/20 °C growth temperature treatment, when all four harvest dates in the continuous growth temperature treatments were averaged (Table 1). Malate and succinate (Figure 1E) also were 50 and 45% less in soybean leaflets when plants were grown in the higher compared to lower temperature treatment. Unlike the other five organic acids shown here, glycerate levels were not temperature responsive (Figure 1D and Table 1). Malonate and fumarate (Figure 1F) were unique in that levels of these two organic acids in the either temperature treatment were similar on the first harvest date (day 0), although both of these compounds were more than 50% lower in the 36/28 compared to the 28/20 °C temperature treatments on the final sampling (day 3).

**Table 1:** Growth temperature treatment effects on organic acids in soybean leaflets. Values are means for data collected one, two and three days after reciprocal temperature shifts between ambient (28/20 °C) and above ambient (36/28 °C) growth temperatures. Probabilities were determined by ANOVA and are for * (*p* ≤ 0.05), ** (*p* ≤ 0.01) and ns, not significant.

Compound	36 °C	36 to 28 °C	28 °C	28 to 36 °C	*p*
	mg g^−1^ DW				
39 Pa CO_2_					
malonate	4.69 ± 0.57	4.54 ± 0.39	6.80 ± 0.57	6.12 ± 0.51	*
glycerate	1.15 ± 0.12	1.25 ± 0.11	1.33 ± 0.16	1.13 ± 0.15	ns
fumarate	0.26 ± 0.07	0.32 ± 0.08	0.36 ± 0.06	0.29 ± 0.05	*
succinate	0.15 ± 0.03	0.16 ± 0.02	0.27 ± 0.04	0.21 ± 0.03	*
malate	5.30 ± 0.43	6.31 ± 0.76	10.66 + 0.85	9.15 ± 0.75	*
citrate	2.17 ± 0.42	4.79 ± 0.53	13.02 ± 1.08	7.92 ± 0.85	**
70 Pa CO_2_					
malonate	7.12 ± 0.86	4.86 ± 0.33	6.98 ± 0.66	6.91 ± 0.74	ns
glycerate	0.67 ± 0.05	0.66 ± 0.06	0.76 ±0 .06	0.92 ± 0.80	*
fumarate	0.65 ± 0.10	0.56 ± 0.10	0.66 ± 0.10	0.80 ± 0.09	ns
succinate	0.13 ± 0.01	0.14 ± 0.01	0.21 ± 0.20	0.18 ± 0.02	*
malate	7.88 ± 0.72	7.33 ± 0.50	12.12 ± 1.30	10.79 ± 1.10	**
citrate	4.99 ± 0.88	5.12 ± 0.69	11.52 ± 1.79	7.97 ± 1.23	**

Transferring whole soybean plants from the 28/20 to the 36/28 °C growth temperature brought about a decrease in all of the temperature responsive organic acids shown in Figure 1 (indicated by dashed lines). Citrate, malate fumarate, succinate and malonate decreased between 60 and 33% over 3 d when compared to similarly aged control plants grown continuously at the lower growth temperature. Transferring soybean plants in the opposite direction, *i.e.*, from the higher to the lower growth temperature, increased levels of citrate, malate and succinate. However, transferring plants from the higher to the lower growth temperature had little effect on fumarate and malonate concentrations. Except for citrate, almost no changes of organic acid concentrations occurred between 0 and 2 d after soybean plants were transferred from the higher to lower growth temperature. Consequently, most of the observed increase took place between 2 and 3 d after the temperature shift. Note that 3 d after a transfer among growth temperature treatments, leaflet concentrations of citrate and malate were intermediate between those of plants kept at the higher and lower growth temperatures.

### 3.2. Growth Temperature Effects on Foliar Organic Acids in the Elevated CO_2_ Treatment

**Figure 2:**
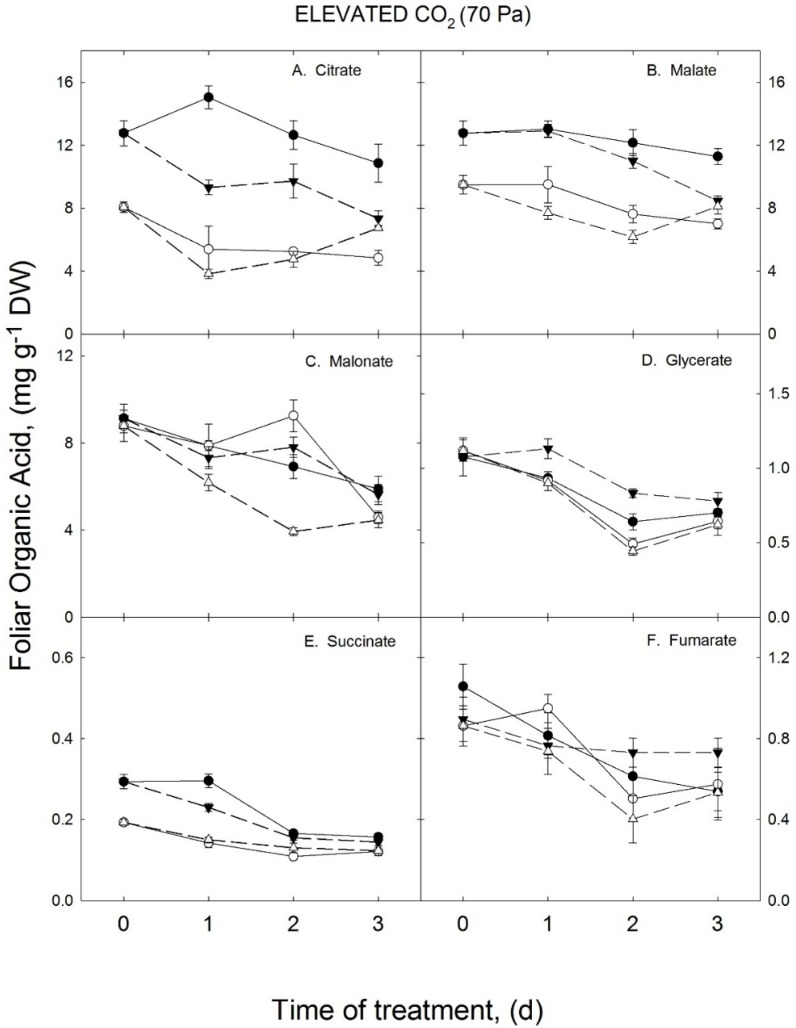
Effects of reciprocal changes of growth temperature on concentrations of organic acids in soybean leaflets under elevated CO_2_ (70 Pa). Experimental details and symbols were as in Figure 1.

It was previously shown that CO_2_ enrichment mitigated the effects of the above optimal growth temperatures on organic acid levels in soybean leaflets (Sicher, 2013). In the current study, citrate and malate (Figure 2A,B) were 54 and 32% lower, respectively, in response to elevated growth temperatures when plants were grown with elevated CO_2_ (70 Pa). Levels of both of these metabolites were greater when grown at 36/28 °C when comparing plants in the elevated to the ambient CO_2_ treatment. As above, these values were obtained by averaging data for the continuous temperature treatments across all four harvest dates. This result confirmed that the effects of high temperature growth conditions on leaflet concentrations of organic acids were lower in elevated than in ambient CO_2_. In contrast to plants grown with ambient CO_2_, changes of foliar malonate and fumarate in response to elevated growth temperatures were not observed in the 70 Pa CO_2_ treatment (Figure 2C,D,F). Succinate differed among growth temperature treatments when plants were exposed to enhanced CO_2_, although this was mostly evident on the first and second harvests (Figure 2E).

Citrate and malate decreased 32 and 25%, respectively, on the final harvest, in response to transferring CO_2_ enriched soybean plants from the lower to the higher growth temperature. There were no visible increases of citrate or malate 2 d after CO_2_ enriched soybean plants were transferred from the higher to the lower growth temperature, although small increases of these two compounds were observed on the 3rd day after temperature treatments were switched. The other four organic acids examined in this study were unaffected by growth temperatures and reciprocally transferring soybean plants among temperature treatments in the presence of elevated CO_2_ had little or no effect on concentrations of these compounds. One other observation was that glycerate levels were lower on all four harvest dates and at both the higher and lower growth temperatures in the ambient compared to the elevated CO_2_ treatment. Unlike the other organic acids measured in this study, this finding suggested that glycerate concentrations in soybean leaflets were diminished by CO_2_ enrichment in both growth temperatures.

**Table 2:** ANOVA results for CO_2_ enrichment effects on organic acids in soybean leaflets. Data were collected on days 0 to 4 using leaflets from plants in continuous temperature treatments and following reciprocal shifts among growth temperature treatments. Probabilities are as in Table 1.

Temperature	Malonate	Glycerate	Fumarate	Succinate	Malate	Citrate
	*p*					
28/20 °C	ns	**	**	ns	**	*
36/30 °C	ns	**	**	ns	ns	**

In addition, the effects of CO_2_ enrichment were determined for organic acid levels in both growth temperature treatments. For this comparison, only samples from the continuous temperature treatments were used (Table 2). Glycerate, fumarate and citrate differed (*p* ≤ 0.05) among CO_2_ treatments regardless of growth temperature but malate was only affected by CO_2_ enrichment when plants were grown with elevated temperatures. Conversely, malonate and succinate were unaffected (*p* > 0.05) by CO_2_ enrichment.

### 3.3. Species Differences in the Effects of Enhanced Growth Temperatures on Foliar Organic Acid Levels

In addition to soybean, growth temperature effects on twelve separate organic acids were determined using pepper, maize and *Phaseolus* leaves (Table 3). Two organic acids, malate and quinate, differed (*p* ≤ 0.05) among the two growth temperature treatments in maize leaves when plants were grown with ambient CO_2_. However, both leaf constituents increased in the enhanced compared to the optimal growth temperature treatment. Malate and citrate also increased (*p* ≤ 0.05) in pepper leaves when plants were grown in the higher compared to the lower growth temperature. In comparison, seven organic acids decreased in common bean leaves when exposed to enhanced growth temperatures. Specific TCA cycle compounds that were decreased by elevated growth temperatures in *Phaseolus* were malate, fumarate and succinate, although citrate did not differ among growth temperature treatments in this species. Taken together, *Phaseolus* and soybean responded similarly, in that several foliar organic acids in both species decreased in response to enhanced growth temperatures.

**Table 3:** Species differences showing effects of supra-optimal growth temperatures on various foliar organic acids. Plants were grown for a minimum of three weeks with 28/20 or 36/28 °C growth temperatures. Sampled leaves were analyzed by a GC-MS procedure and values in bold print within species differed at *p* ≤ 0.05.

Compound	*Zea Mays*		*Capsicum Annum*		*Phaseolus Vulgaris*	
	28/20 °C	36/28 °C	28/20 °C	36/28 °C	28/20 °C	36/28 °C
	μg g^−1^ DW					
Maleic	141	179	5184	3277	297	**89**
Malic	9886	**13612**	7292	**11090**	37570	**9850**
Adipic	179	133	5040	4323	205	172
Quinic	104	**217**	3323	3805	40	19
2-Oxoglutaric	ND	ND	ND	ND	56	37
Aconitic	39075	36984	63	63	54	**23**
Shikimic	1008	827	202	195	86	102
Citric	1189	904	7539	**12133**	12043	11682
Malonic	55	56	944	583	6991	**329**
Glyceric	2898	2981	2833	2211	2667	**1912**
Fumaric	32	56	28	33	288	**49**
Succinic	83	95	69	100	403	**144**

## 4. Discussion

Organic acids perform vital functions in respiratory, photorespiratory and photosynthetic metabolism, and these compounds are normally synthesized from the oxidation of soluble sugars and lipids [18,19]. Organic acids serve as substrates for amino acid and secondary product formation and also shuttle protons among subcellular compartments to manage energy balance and cellular pH. A number of important organic acids function in the TCA cycle, which performs a central role in oxidative cellular metabolism. Abiotic stress greatly affects levels of specific soluble carbohydrates and amino acids in plant tissues that normally function as osmolytes, compatible solutes and as scavengers for reactive oxygen species [1,2,3]. In comparison, for the above compounds, much less is known about the role of organic acids in responding to abiotic stress.

In a previous study, supra-optimal growth temperatures drastically reduced concentrations of specific TCA cycle components (*p* ≤ 0.05) in soybean leaflets [11]. This finding was confirmed in the present study and, in addition, this process was only partially reversed 3 d after soybean plants in the ambient CO_2_ treatment were transferred from higher to lower growth temperatures. Similar conclusions applied to the reverse experiment in which plants were transferred from the lower to higher growth temperature. In almost every instance, a shift from lower to higher temperature brought about a slightly more rapid and larger decrease in foliar organic acid content than the reverse temperature shift. This result emphasized the continuing impact of elevated growth temperatures on organic acid levels after high temperature treatments ended. Note that in our prior study [11], single leaf rates of P_N_ were increased by 20% when plants were grown using the 36/28 compared to the 28/20 °C growth temperature treatment. Consequently, decreased organic acid levels in soybean leaflets in response to elevated growth temperatures and with ambient CO_2_ were not likely the result of a temperature inhibition of P_N_. Note that organic acid levels in leaves would be expected to change within less than 1 d if the effects of enhanced growth temperatures were solely attributable to an inhibition of P_N_. Therefore, it is likely that the synthesis of TCA cycle intermediates was impaired in soybean plants treated with elevated growth temperatures.

Elevated temperature effects on malonate and fumarate concentrations were minimal on day 0, when plants were grown with ambient CO_2_. However, diminished concentrations of both of these compounds were observed 3 or 4 d later when plants were grown with supra-ambient temperatures. This suggested that temperature effects on levels of these two organic acids in soybean leaflets were affected by leaf age. Both growth temperature and CO_2_ enrichment affect rates of whole plant development and leaf appearance rates. In the current study, sampling was initiated 1 d earlier in the higher compared to the lower growth temperature to compensate for differing developmental rates among treatments. However, it remains likely that differences in leaf development affected organic acid measurements in these experiments. Changes of leaf development also could explain some of the differences in organic acid levels observed in plants shifted from one temperature treatment to the other. Large quantities of foliar organic acids are stored in the vacuole and it is possible that this organelle is involved in the temperature dependent changes of organic acids observed in the current study.

In agreement with an earlier report [11], CO_2_ enrichment mitigated the effects of enhanced growth temperatures on organic acids in soybean leaflets. Unlike when using ambient CO_2_, malonate and fumarate did not differ among growth temperature treatments when soybean plants were grown with elevated CO_2_. In addition, citrate and malate concentrations in leaflets from the 36/28 °C temperature treatment were greater in the 70 compared to the 39 Pa CO_2_ treatment. Glycerate, which is involved in lipid metabolism and it is transported into the chloroplast in exchange for glycolate during the terminal steps of the photorespiratory pathway [19], decreased in soybean leaflets in response to CO_2_ enrichment at both growth temperatures. According to Sharkey [20] rates of P_R_ can achieve half the rate of P_N_ depending upon environmental conditions. Therefore, it is possible that elevated CO_2_ treatments inhibited photorespiration, which decreased foliar glycerate levels in the 36/28 °C temperature treatment.

Based on the above discussion, it is possible that the depletion of various organic acids in soybean leaves exposed to elevated growth temperatures, and the mitigation of this change attributed to CO_2_ enrichment, also involved temperature and CO_2_ effects on rates of photorespiration. A possible explanation may be that enhanced flux through P_R_ resulted in increased glycine decarboxylase activity. This enzyme is a major component of mitochondria in green leaves where it converts two molecules of glycine to serine, CO_2_ and NADH [21]. Excess NADH levels in foliar mitochondria would impede glycolytic flux by feedback inhibition of the pyruvate dehydrogenase carboxylase and this would down regulate the entire TCA cycle [22]. This explanation is consistent with known effects of enhanced temperatures on photorespiration and their reversal by CO_2_ enrichment. However, it was unexpected that the reversal of depleted organic acid levels in soybean leaflets by CO_2_ enrichment or by a transfer to lower growth temperatures took more than 3 d to complete. This suggested that, in addition to effects on P_R_, high growth temperatures impaired important metabolic pathways in soybean leaflets [23]. It is also clear from temperature transfer experiments that this damage was difficult to reverse in recently expanded soybean leaflets.

Experiments with differing plant species showed that malate, citrate or quinate increased in either maize or pepper leaves from plants grown with supra-optimal temperatures. Conversely, there were large declines in seven out of twelve organic acids in *Phaseolus* leaves in response to enhanced growth temperatures. The finding that organic acid responses to elevated growth temperatures were similar in soybean and *Phaseolus* suggested that high temperature effects on foliar organic acids may be in concentrated in legume species. Observations with maize and pepper leaves supported prior findings by Yu *et al.* [14] showing that citrate increased in turf grass species exposed to high temperatures.

Current findings confirmed an earlier report [11] that foliar organic acids, and particularly those associated with the TCA cycle, decreased 40 to 80% in response to supra-optimal growth temperatures. Enhanced temperature effects on organic acids in soybean leaflets were only partially reversed 3 d after growth temperatures were lowered. In addition, four of six organic acids, including glycolate, in this study were altered by CO_2_ enrichment when plants were raised in both optimal and enhanced temperature treatments. The current study identified three factors that potentially contributed to diminished organic acid levels in soybean leaflets exposed to elevated growth temperatures. These were the following: (1) an impairment of metabolic pathways responsible for organic acid synthesis, (2) the combined effects of elevated temperature and CO_2_ enrichment on P_R_, and (3) temperature and CO_2_ effects on leaf development. Findings here also suggested that temperature effects on foliar organic acids were more evident in legumes than in non-leguminous species.

## Supplementary Files

Supplementary File 1
